# Orelabrutinib Combined With Lenalidomide and Immunochemotherapy for Relapsed/Refractory Primary Central Nervous System Lymphoma: A Retrospective Analysis of Case Series

**DOI:** 10.3389/fonc.2022.901797

**Published:** 2022-06-16

**Authors:** Chuanwei Yang, Yong Cui, Xiaohui Ren, Ming Li, Kefu Yu, Shaoping Shen, Haihui Jiang, Mingxiao Li, Xiaokang Zhang, Xuzhe Zhao, Qinghui Zhu, Song Lin

**Affiliations:** ^1^ Department of Neurosurgery, Beijing Tiantan Hospital, Capital Medical University, Beijing, China; ^2^ Beijing Neurosurgical Institute, Capital Medical University, Beijing, China; ^3^ Department of Neurosurgery, Henan Provincial People's Hospital, People's Hospital of Zhengzhou University, Zhengzhou University, Henan, China; ^4^ Department of Pharmacy, Beijing Tiantan Hospital, Capital Medical University, Beijing, China; ^5^ Department of Neurosurgery, Peking University Third Hospital, Peking University, Beijing, China; ^6^ National Clinical Research Center for Neurological Diseases, Center of Brain Tumor, Beijing Key Laboratory of Brain Tumor, Beijing Institute for Brain Disorders, Beijing, China

**Keywords:** Orelabrutinib, safety, efficacy, relapsed/refractory, primary central nervous system lymphoma, genomic characteristics

## Abstract

**Background:**

Relapsed/refractory (r/r) primary central nervous system lymphoma (PCNSL) is an intractable situation without sound treatment. Bruton’s tyrosine kinase (BTK) represents an attractive drug target in PCNSL. Orelabrutinib is a new-generation BTK inhibitor with high cerebrospinal fluid (CSF) concentration. This study aimed to evaluate the efficacy and safety of orelabrutinib-containing combination therapy in patients with r/r PCNSL.

**Methods:**

We retrospectively analyzed r/r PCNSL patients who received combination therapy with rituximab, high-dose methotrexate, temozolomide, orelabrutinib and lenalidomide, and further explored the relationship between the efficacy and genetic characteristics.

**Results:**

A total of fifteen patients were included in this retrospective study. The overall response rate (ORR) was 86.7%, the complete remission (CR) rate was 73.3% and the disease control rate (DCR) was 93.3%. Among 13 responders, 9 patients are still receiving oral orelabrutinib and lenalidomide. The most common adverse event (AEs) was transaminase increase (66.7%). No grade 4 AE or drug-related death was reported. Genomic sequencing showed that patients who responded to orelabrutinib had abnormal NF-κB activation, while those who had no response were mainly enriched with transcriptional misregulation. Patients who had mutations in TLR, BCR, or NF-κB pathway achieved complete or partial response to the orelabrutinib-containing therapy. Moreover, the blood and cerebrospinal fluid circulating tumor DNA (ctDNA) were closely associated with tumor recurrence and treatment response and sustained tumor responses correlated with the clearance of ctDNA.

**Conclusion:**

Orelabrutinib-containing regimen was effective and well-tolerated in patients with r/r PCNSL. Genome sequencing of tumor samples could help to screen patients who may respond to the orelabrutinib-containing regimen, and liquid biopsy may contribute to tracing tumor burden and monitoring treatment response.

## Introduction

Primary central nervous system lymphoma (PCNSL) is a rare and aggressive subtype of non-Hodgkin’s lymphoma, confining to the central nervous system (CNS) or eyes without systemic involvement. PCNSL incidence is approximately 5 per million and has been gradually increasing in recent decades (1). Approximately 95% of PCNSL pathology is represented by diffuse large B-cell lymphoma (DLBCL) (2). Compared to other extranodal DLBCL, PCNSL is associated with a relatively poor prognosis, with a 5-year survival rate of only 20%-30% (1). High-dose methotrexate (HD-MTX)-based chemotherapy has been recommended as the first-line treatment, however, about 30% of PCNSL cases are refractory to the HD-MTX-based chemotherapy, and up to 60% will eventually relapse (3–6). Moreover, about 25% of relapsed PCNSL cases fail to respond to the initial treatment (7). It was reported that the median survival for relapsed/refractory (r/r) PCNSL was only 8-18 months despite intensive treatment (3). Few effective treatment options and novel therapeutics exist for patients with refractory and early relapsed PCNSL, and thus a standard of care remains to be established. Although guidelines recommend whole-brain radiotherapy (WBRT) or autologous stem cell transplantation (ASCT), the usage is limited due to the late neurotoxicity associated with radiotherapy and the high mortality of myeloablative chemotherapy prior to ASCT. Notably, targeted therapy and immunochemotherapy may play an important role in this situation (8).

Compared to DLBCL without CNS, PCNSL has its unique genomic signature. Myeloid differentiation factor 88 (*MYD88*, 60%-80%) and *CD79B* (50%-60%) are more frequently mutated in PCNSL, and present not only in inactivated B cell-like-DLBCL (ABC-DLBCL) but also in germinal B cell-like-DLBCL (GCB-DLBCL) (9–11). *MYD88* L265P and *CD79B* Y196 are the two most common gain-of-function mutations in PCNSL, which are parts of toll-like receptors (TLR) and B-cell receptor (BCR) signaling pathways, respectively (10–12). The mutation of *MYD88* and *CD79B* was also used to classify DLBCL into a MCD subtype with a poor prognosis (13). Bruton tyrosine kinase (BTK) mediates the nuclear factor (NF)-κB signaling pathway downstream of TLR and BCR. Activation of NF-κB signaling continues to provide survival signals to tumor cells. Therefore, BTK plays an important role in maintaining the malignant phenotype of PCNSL and is an attractive drug target in PCNSL.

The first-generation BTK inhibitor ibrutinib has shown antitumor activity in r/r PCNSL patients as a single agent or combination treatment (14, 15). Although ibrutinib revolutionized the treatment of B cell malignancies, its use has been limited by serious adverse events (AEs), such as fungal infections and atrial fibrillation resulting from off-target inhibition (15, 16). This has led to the development of highly selective BTK inhibitors with fewer off-target effects. Orelabrutinib, a new generation of BTK inhibitor, not only has higher blood-brain barrier permeability and higher bioavailability but also has excellent kinase selectivity and little off-target side effect (17). It has been approved by National Medical Products Administration for the treatment of mantle cell lymphoma and chronic lymphocytic leukemia. Results of several studies have demonstrated the anti-tumor activity and manageable safety profile of orelabrutinib (18, 19). Besides, tirabrutinib as a BTK inhibitor was approved for the treatment of PCNSL (20). More importantly, per-clinical data showed the synergistic effect of orelabrutinib as a partner for combination therapy (21). Therefore, we conducted a retrospective analysis of 15 patients with r/r PCNSL to evaluate the efficacy and safety of combination therapy with rituximab, high-dose methotrexate (HD-MTX), and temozolomide (RMT), as well as orelabrutinib and lenalidomide. Additionally, we also explored genetic differences between responders and non-responders to orelabrutinib and the changes in circulating tumor DNA (ctDNA) in blood and cerebrospinal fluid (CSF) before and during the treatment.

## Materials and Methods

### Patients

Adult immunocompetent consecutive patients with r/r PCNSL who have received the combination therapy with RMT, orelabrutinib, and lenalidomide were retrospectively analyzed at the Department of Neurosurgery, Beijing Tiantan Hospital between October 2020 and February 2022. The diagnosis of the CNSL and diffuse large B-cell lymphoma subtype (non-germinal center B-cell-like [non-GCB], GCB, unknown) was made according to the Lymphoid Malignancies Guidelines 2021, which were established by the Chinese Society of Clinical Oncology (22). Relapsed lymphoma was defined as lymphoma that relapsed after the complete response (CR) to prior treatment (23).

According to the NCCN Guidelines for Central Nervous System (CNS) Cancers, the diagnosis of PCNSL was made according to the following findings: 1) Diffuse large B-cell lymphoma (DLBCL) confirmed by stereotactic biopsy of brain lesions; 2) No invasion outside the CNS confirmed by physical examination (PET and CT examination, ophthalmic examination, and bone marrow aspiration and biopsy). Refractory lymphoma was diagnosed if any of the following criteria were met: the tumor shrank < 50% or progressive disease after 4 courses of chemotherapy; CR was achieved by standard chemotherapy but relapsed within 6 months; there were two or more relapses after CR; and relapse after hematopoietic stem cell transplantation (24). The inclusion criteria were as follows: age ≥ 18 years, a physician’s diagnosis of r/r PCNSL, and receiving orelabrutinib combination regimens. Patients with mental disorders, unstable systemic disease, immunodeficiency, or pregnancy were excluded. This retrospective study was approved by the Medical Ethics Committee of the Beijing Tiantan Hospital (Ethics Approval No. YW2020-038-02) and was conducted in accordance with the principles of the Declaration of Helsinki.

### Treatment Regimen

All patients had received the combination regimens, including rituximab, HD-MTX, temozolomide, orelabrutinib, and lenalidomide. Rituximab (dosed at 375 mg/m^2^) was administered intravenously (IV) on day 1, followed by HD-MTX (dosed at 3.5 g/m^2^) over 4 hours on day 2. In order to reduce toxicity, approximately 24 hours after chemotherapy, leucovorin (50 mg) was injected intramuscularly every 6 hours until the MTX plasma concentration decreased to 0.05 μmol/L. Temozolomide was administered at a dose of 150 mg/m^2^ once daily on days 4 to 8. The rituximab, HD-MTX, and temozolomide (RMT) regimen were administrated every 4 weeks. Oral orelabrutinib (150 mg once a day) and lenalidomide (25 mg once a day) along with prednisone (10 mg once a week) were administered until disease progression, death, or intolerable toxicity. The maximum duration of the RMT regimen was decided by the clinicians according to the patient’s disease condition.

### Assessment

Tumor responses were evaluated using magnetic resonance imaging (MRI), positron emission tomography (PET), CSF examination, ocular slit lamp, and bone marrow biopsy, according to the International PCNSL Collaborative Group (IPCG) guidelines (17). MRI evaluation was performed every cycle, and the time interval was gradually extended to 3-6 cycles after the patient reached a CR. Total tumor volume was the sum of volumes of lesions calculated by the largest longitudinal diameter multiplied by its perpendicular diameter on the MRI image. The efficacy endpoints were overall response rate (ORR), disease control rate (DCR), progression-free survival (PFS), and overall survival (OS). ORR was defined as the proportion of patients who achieved partial response (PR) and CR. DCR was defined as the proportion of patients who achieved PR, CR, and stable disease (SD). PFS was calculated from the initiation of treatment to the time of disease progression or death. OS was defined as the time from treatment to death due to any cause.

Safety and tolerability were evaluated through physical examination, vital signs, laboratory tests (including urinalysis, hematology, and blood chemistry), electrocardiography, and adverse events (AEs). AEs were collected and assessed following the Common Terminology Criteria for Adverse Events (CTCAE) version 5.0.

### Sample Collection and Genomic Analysis

To explore the association between genomic characteristics and tumor response to orelabrutinib, we collected available baseline tumor biopsy samples from patients treated with orelabrutinib for genomic sequencing. Besides, all blood and CSF samples were collected before the RMT therapy and after 2 cycles of orelabrutinib and lenalidomide therapy. Archived blood samples were used for gene mutation assessments of *MYD88 L265P* and *CD79B Y196H* using the droplet digital polymerase chain reaction (ddPCR) or the next-generation sequencing (NGS).

### Statistics Analysis

Statistical analyses and visualization were performed using R 4.0.0 and GraphPad Prism version 6.0 statistical software. Continuous variables were expressed as means (standard deviations) and medians (range); Categorical variables were expressed as numbers (proportions). The PFS and OS were estimated using the Kaplan-Meier method with 95% confidence intervals (CIs). A two-sided *p*<0.05 was considered statistically significant.

## Results

### Patients’ Characteristics

Between October 2020 and February 2022, a total of 15 eligible patients with r/r PCNSL (5 males, 10 females) were included in this retrospective study (**Table 1**), with a median age of 62 years (range, 33 to 78 years) and a median Karnofsky performance score of 60 (range, 40-90). Of these, 10 patients (66.7%) were relapsed disease and 5 (33.3%) were refractory to HD-MTX-based chemotherapy. Regarding the histological subtypes of PCNSL, there were 4 (26.7%) patients with GCB and 11 (73.3%) patients with non-GCB. Nine (60%) patients had tumors involving the deep brain, including the periventricular tissue, basal ganglia, corpus callosum, brainstem, and/or cerebellum. None of the patients received ASCT or radiotherapy before treatment, while 3 patients (P4, P5, and P6) received WBRT after treatment. Of all included patients, 5 had previously received orelabrutinib therapy; 4 (P1, P2, P3, and P15) of whom had a PR to orelabrutinib therapy and 1 (P4) had a progressed disease (PD). Seven of the 15 patients had previously received the RMT regimen. The median number of prior regimens was 2 (range, 1-5); and 3 patients (P1, P4, and P5) have received ≥3 prior lines. The median cycle of RMT therapy was 1 (range, 1-5). 9 patients received 1 cycle of RMT treatment and 6 patients received ≥2 cycles. The median follow-up time was 219 days (range, 62-498).

**Table 1:** Baseline characteristics of the relapsed/refractory PCNSL patients.

Characteristics	Patients (n = 15)
Age-years, median (range)	62 (33-78)
Median KPS score (range)	60 (40-90)
Sex, n (%)	
Male	5 (33.3)
Female	10 (66.7)
PCNSL subtype n (%)	
GCB	4 (26.7)
Non-GCB	11 (73.3)
Tumor location n (%)	
Superficial brain	6 (40)
Deep brain	9 (60)
Median number of prior regimens (range)	2 (1-5)
Median cycle of RMT salvage therapy (range)	1 (1-5)

PCNSL, primary central nervous system lymphoma; GCB, germinal B cell-like; RMT, rituximab, high-dose methotrexate, and temozolomide.

### Efficacy

**Figure 1:**
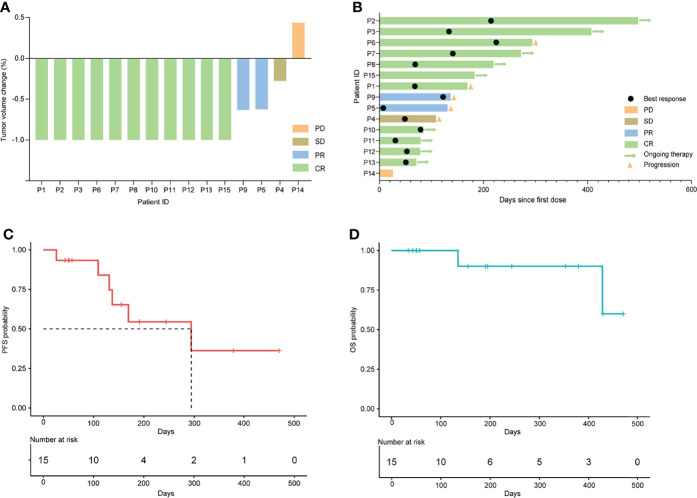
Clinical response to orelabrutinib-containing combination therapy in r/r PCNSL. **(A)** Tumor volume change between best response and baseline. **(B)** Best response and the duration of the combination therapy. **(C)** Kaplan-Meier analysis for progression-free survival. **(D)** Kaplan-Meier analysis for overall survival.

**Figure 2:**
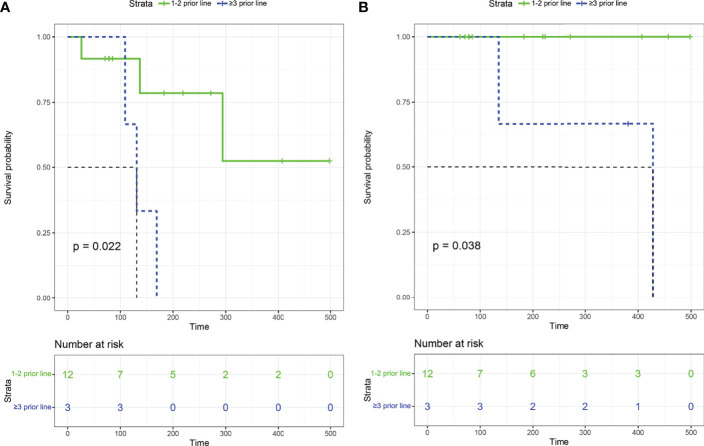
Kaplan–Meier survival curves among patients with 1-2 prior lines and ≥3 prior lines. **(A)** Progression-free survival. **(B)** Overall survival.

All the 15 eligible patients were included for efficacy analysis. The best responses were achieved, including 11 CR (11/15, 73.3%), 2 PR (2/15, 13.3%), 1 SD (1/15, 6.7%, P4) and 1 PD (1/15, 6.7%, P14), which resulted in an ORR of 86.7% (13/15) and DCR of 93.3% (14/15). The best changes in tumor diameter from baseline to the study closing date are shown in **Figure 1A**. Among these responders, 9 patients are still receiving orelabrutinib and lenalidomide therapy (**Figure 1B**). The median TTR was 23 days (range, 4-54 days), and the median time to best response was 66 days (range, 6-224 days). In addition, of 9 patients treated with 1 cycle of RMT, the best responses after this treatment regimen were 6 CR (66.7%), 1 PR (11.1%), 1 SD (11.1%), and 1 PD (11.1%), respectively; of 6 patients received ≥2 cycles of RMT, 5 (83.3%) achieved a CR and 1 (16.7%) achieved a PR after this treatment regimen. The median PFS was 9.8 months (294 days, 95% CI: 137-NA) for the cohort (**Figure 1C**). Two patients (P4 and P5) died due to tumor progression. The median OS was not reached (**Figure 1D**). Patients who received 1-2 prior lines showed a longer PFS (p=0.022; **Figure 2A**) and OS (p=0.038; **Figure 2B**) than these received ≥3 prior lines. The treatment regimens and disease progression of each patient were summarized in **Table S1**.

**Figure 3:**
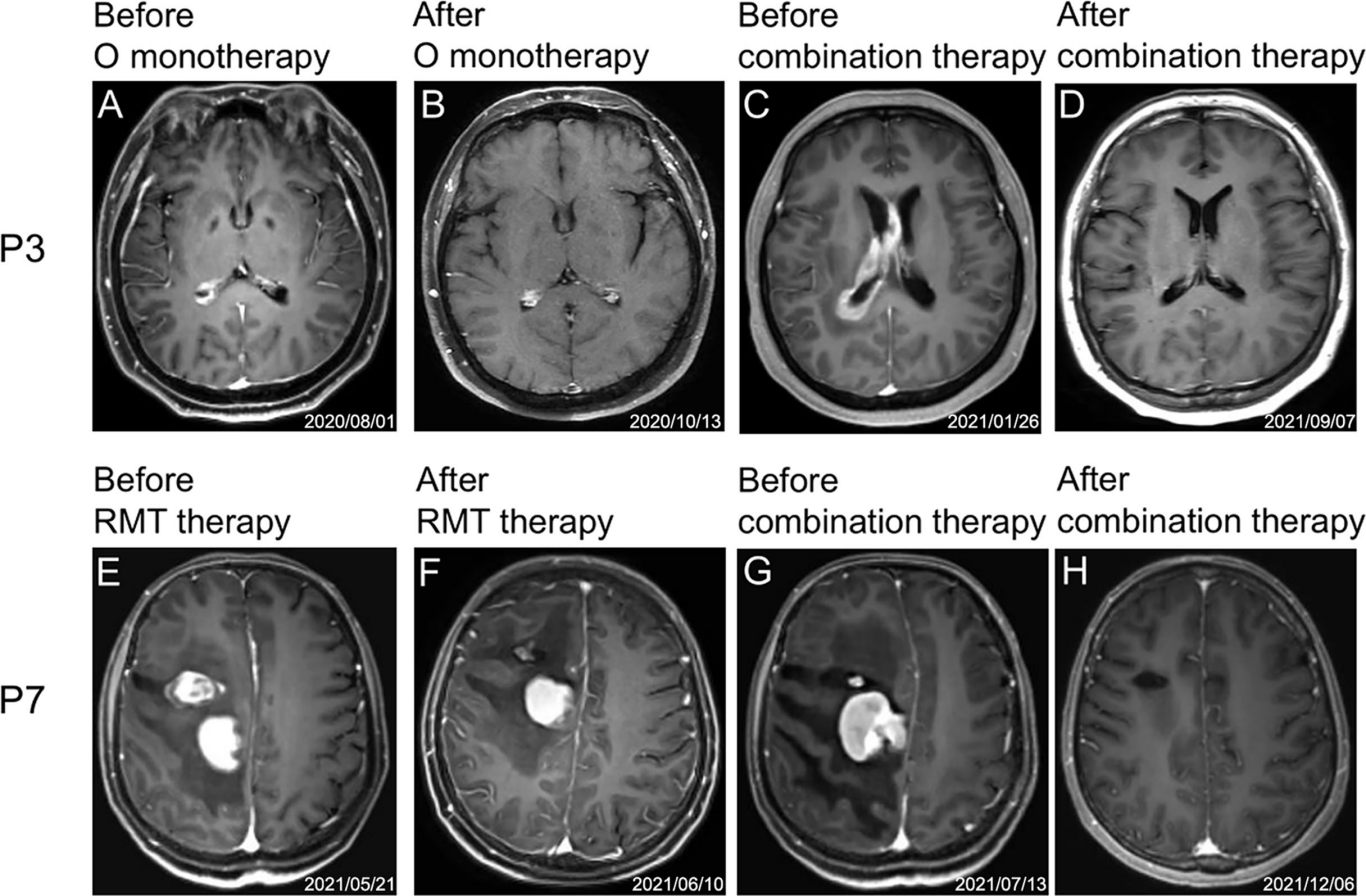
Representative MRI images of two patients (P3 and P7) during treatment. **(A–D)** and **(E–H)** are MRI images of the treatment process of P3 and P7, respectively. **(A)** Before orelabrutinib therapy. **(B)** After orelabrutinib therapy. **(C)** Before combination therapy. **(D)** After combination therapy. **(E)** Before RMT therapy. **(F)** After RMT therapy. **(G)** Before combination therapy. **(H)** After combination therapy.

Three patients (P4, P5, and P6) received etoposide or programmed death (PD)-1 antibody due to the excessive tumor burden, and patient P4 also received chidamide (the selective inhibitor of histone deacetylase). The best response of these 3 patients was SD (P4), PR (P5), and CR (P6), respectively. Notably, 2 patients (P7 and P9) experienced the regimen adjustment by replacing the lenalidomide with temozolomide due to due to hypersensitivity to lenalidomide. As a result, P7 still achieved and maintained CR, and P9 achieved PR. None of the patients received antifungal prophylaxis treatment. Of the 5 patients receiving prior orelabrutinib treatment, 4 achieved CR(P1, P2, P3, P15)and 1 (P4) achieved SD after orelabrutinib-containing combination therapy (**Figure 3**). Of the 7 RMT prior treated patients, 4 achieved CR (P1, P7, P8, P12), 2 PR (P5, P9) and 1 PD (P14) (**Figure 3**).

### Clinical Response and Genomic Characteristics

**Figure 4:**
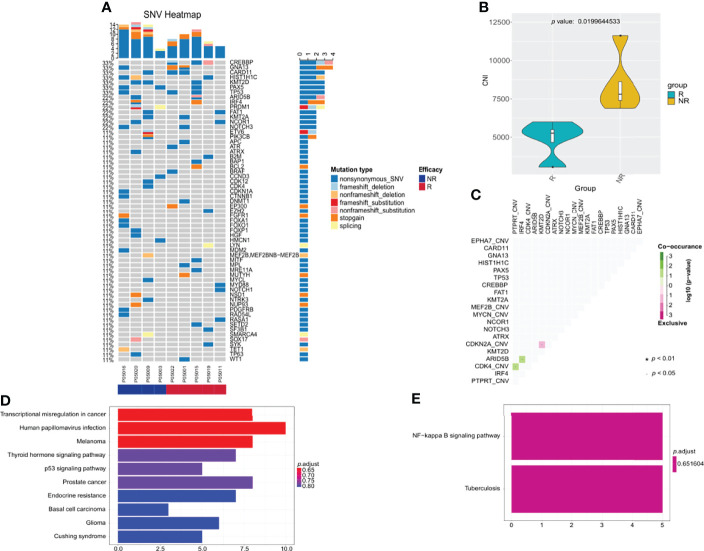
The relationship between clinical response and genetic characteristics in 9 patients treated with orelabrutinib therapy. **(A)** The gene mutation spectrum. **(B)** Significant differences showed between responders and non-responders in copy number instability (CNI), and non-responders had much higher CNI. **(C)** Analysis of gene co-mutation and mutual exclusion. **(D)** Gene enrichment analysis of non-responders. **(E)** Gene enrichment analysis of responders.

Baseline tumor biopsy samples were available for 9 patients receiving orelabrutinib therapy. Of these, 5 patients were orelabrutinib-responders with the best response of PR, and 4 were non-responders with the best response of SD (1 patient) or PD (3 patients). We explored the association between treatment response and tumor gene mutation. As shown in **Figure 4A**, the most frequently mutated genes were *CREBBP*, *GNA13*, *CARD11*, *HIST1H1C*, *KMT2D*, *PAX5*, and *TP53* in this cohort. Additionally, patients who had *CARD11* mutation also responded to orelabrutinib. There were no significant differences in efficacy between different ages, genders, pathological subtypes, or tumor sites. However, significant differences were observed in genomic traits between orelabrutinib-treated patients in different response groups; that is, non-responders had much higher copy number instability (CNI) score, indicating that non-responders had a higher degree of chromosomal instability (**Figure 4B**). Mutual exclusion analysis of gene co-mutation showed that *IRF4*, *ARID5B* and *CDK4*, *PTPRT* were co-occurrent mutations, while *CDKN2A*, *KMT2D* were exclusive mutations in this cohort (**Figure 4C**).

**Figure 5:**
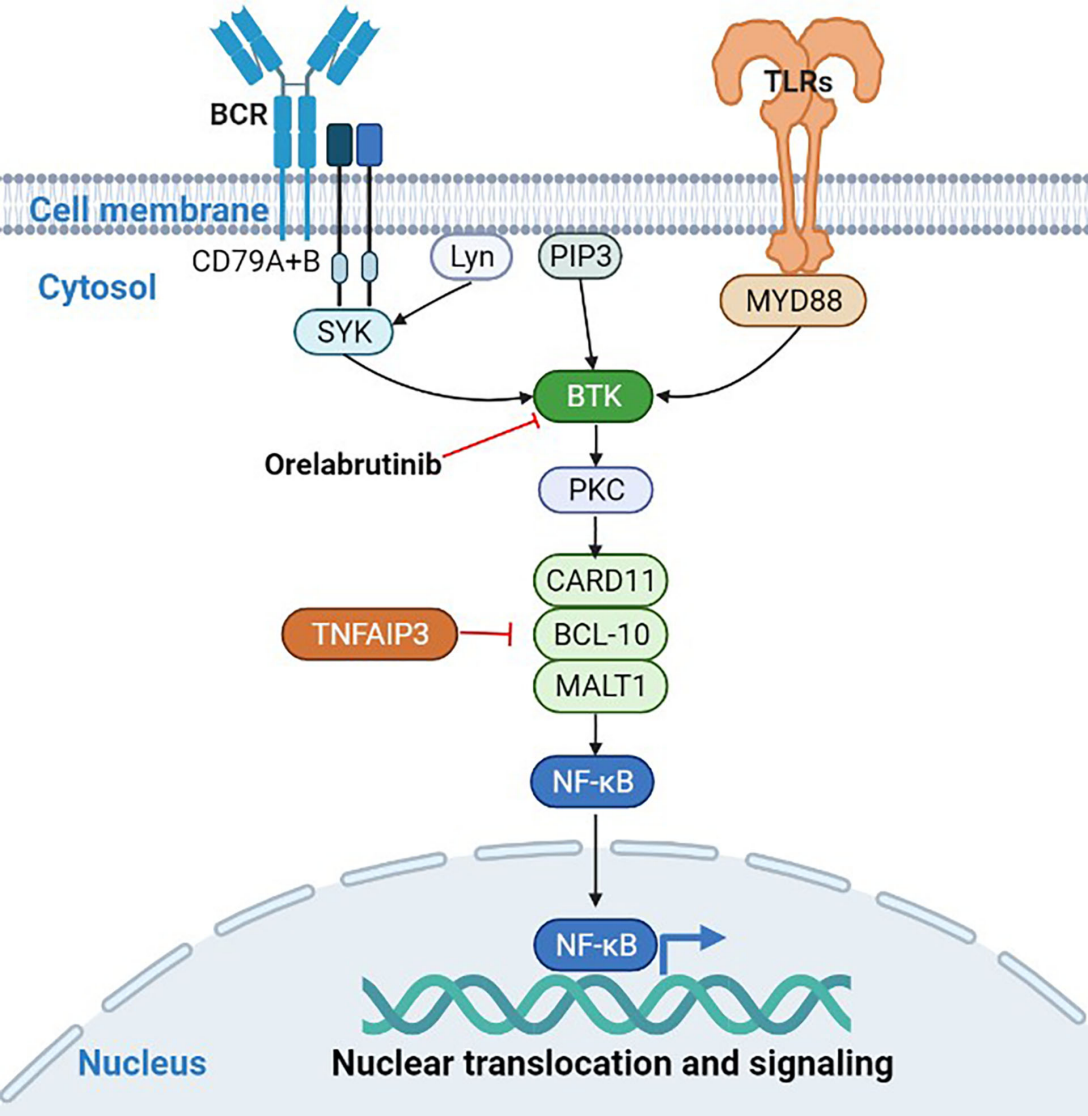
Signaling pathways involved in the mechanisms of orelabrutinib.

Gene enrichment analysis demonstrated that the mutated genes in non-responders were mainly enriched in transcriptional misregulation pathways (**Figure 4D**), while the mutated genes in responders were mainly enriched in NF-κB abnormally activated pathways (**Figure 4E**). As shown in **Table 2**, patients who had gene mutations in the TLR pathway (*MYD88*), BCR pathway (*CD79B*, *CARD11*, *TNFAIP3*), NF-κB pathway (*PIM1*, *IRF4*, *BTG2*), and cell cycle pathways (*TP53*, *CDKN2A*) achieved better response (CR or PR) to the orelabrutinib-containing combination therapy, while patients without the above gene mutations achieved a poor response. PCNSL that relies on the activation of the BCR or TLR signaling pathway to activate the NF-κB signaling pathway has a better response to the orelabrutinib-containing combination therapy. The mechanism of action of BTK inhibitor orelabrutinib is summarized in **Figure 5**.

**Table 2:** Gene mutations in pathways in pretreatment tumor tissue.

Id	Disease	Status	Subtype	Response	TLR pathway	BCR pathway	NF-κB pathway	Cell cycle pathway
P1	PCNSL	Relapsed	non-GCB	CR	NA	NA	NA	NA
P2	PCNSL	Refractory	non-GCB	CR	WT	*CD79B*	PIM1	CDKN2A
P3	PCNSL	Relapsed	GCB	CR	WT	*CD79B*、CARD11	WT	TP53
P4	PCNSL	Relapsed	non-GCB	SD	WT	WT	WT	WT
P5	PCNSL	Relapsed	GCB	PR	NA	NA	NA	NA
P6	PCNSL	Relapsed	GCB	CR	WT	TNFAIP3、	WT	TP53
P7	PCNSL	Relapsed	non-GCB	CR	*MYD88*	*CD79B*	PIM1IRF4	WT
P8	PCNSL	Relapsed	non-GCB	CR	WT	WT	BTG2	WT
P9	PCNSL	Refractory	non-GCB	PR	*MYD88*	*CD79B*	PIM1	CDKN2A
P10	PCNSL	Relapsed	non-GCB	PR	WT	*CD79B*CARD11	PIM1、BTG2、IRF4	WT
P11	PCNSL	Refractory	non-GCB	PR	WT	WT	BTG2	WT
P12	PCNSL	Relapsed	non-GCB	PR	*MYD88*	*CD79B*	PIM1	WT
P13	PCNSL	Refractory	GCB	PR	NA	NA	NA	NA
P14	PCNSL	Refractory	non-GCB	PD	WT	WT	WT	TP53
P15	PCNSL	Relapsed	non-GCB	CR	*MYD88*	*CD79B*	PIM1、IRF4	TP53、CDKN2A

PCNSL, primary central nervous system lymphoma; GCB, germinal B cell-like; CR, complete remission; PR, partial remission; SD, stable disease; PD, progression disease; NA, not available; WT, wild-type; TLR, Toll-like receptor; BCR, B-cell receptor; NF-κB, nuclear factor-kappa B.

### Disease Surveillance Through ctDNA

In the combination therapy, the ddPCR detection of *MYD88* and *CD79B* mutations was performed in 9 patients using blood samples and the NGS was performed in 1 patient using CSF. Among these patients, 3 patients (P7, P9, P15) showed *MYD88* or *CD79B* positive before the combination therapy and turned negative after two cycles of the combination therapy. Two patients (P7 and P9) showed no system involvement. P15 showed gastric involvement which was confirmed DLBCL two months later by biopsy. Additionally, one patient (P6) was detected as negative by blood ddPCR and negative CSF cytology, but positive in the next-generation sequencing of CSF. However, the tumor relapses about two months later, and the blood ddPCR detection was still negative at that time. The mutational genes in CSF were consistent with that of his baseline tumor biopsy sample.

### Safety

The overall incidence of AEs was summarized in **Table 3**. 10 patients (66.7%) experienced treatment-related AEs. Most common treatment-related AEs of any grade were transaminase increase (10/15, 66.7%), fatigue (6/15, 40%), and leukopenia (4/15, 26.7%). The reported grade 3 or worse AE was fatigue (6, 40%). Two patients (13.3%) had grade 3 hypersensitivity reactions and diarrhea to lenalidomide, respectively, and had resolution of symptoms after withdrawal of lenalidomide. No other patients required lenalidomide dose reduction. Most grade 2 or fewer toxicities were transaminase increase (66.7%), leukopenia (26.7%), and drowsiness (20%). No fungal infection was observed. Reported AEs were generally manageable and resolved soon after supportive treatment. No grade 4-5 toxicity was reported.

**Table 3:** Adverse events of Orelabrutinib-containing therapy.

Adverse event	Grade 1	Grade 2	Grade 3	Total (%)
Hematological toxicities				
Leukopenia	3	1		4 (26.7%)
Neutropenia		1		1 (6.7%)
Purpura	1			1 (6.7%)
Non-hematological toxicities				
Transaminase increase	6	4		10 (66.7%)
Fatigue			6	6 (40%)
Drowsiness		3		3 (20%)
Diarrhea		1		1 (6.7%)
Constipation	1			1 (6.7%)

RMT, rituximab, high-dose methotrexate and temozolomide; OL, orelabrutinib and lenalidomide.

## Discussion

This study is the first report of orelabrutinib, lenalidomide plus immunochemotherapy in r/r PCNSL. In this study, the use of this combination regimen showed inspiring antitumor activity with ORR of 86.7% and DCR of 93.3%, as well as acceptable toxicity in r/r PCNSL patients. Our findings supported the clinical application of this combination regimen in r/r PCNSL, which could provide a promising therapeutic strategy for this patient population.

The present study demonstrated an encouraging result in r/r PCNSL. The ORR achieved with orelabrutinib-containing regimen was 86.7%, which was comparable with other BTK inhibitor-containing regimens, such as results from ibrutinib (ORR, 52%) (25), temozomide/ibrutinib (ORR, 55%) (26), and the phase I/II study of tirabrutinib (ORR, 64%) (27). Nevertheless, a phase II showed that lenalidomide plus rituximab without orelabrutinib only induce an ORR of 35.6% (28). These results supported that various BTK inhibitors are potent therapeutic options against r/r PCNSL and especially adding orelabrutinib may provide additional benefit for this population. Actually, we observed a relative high ORR and DCR in this study. As a preliminary study reported, the favorable blood-brain barrier permeability of orelabrutinib could induce a high CSF concentration of 20.10 ± 14.70 ng/mL (29), which may partly explain the better response in these patients. Besides, the synergistic effects of BTK inhibitor and Combination of drugs may be another reason for better response, such as the combination of orelabrutinib and rituximab (21), BTK inhibitor and lenalidomide (30). The combination therapy eliminates tumor cells from multiple aspects by killing tumor cells directly with chemotherapy, blocking proliferation pathways with targeted therapy, and modulating the tumor microenvironment with immunotherapy. Besides, there were no treated-death or intolerable toxicity after combination therapy, which induced the better medication compliance. Most importantly, tumor load was reduced quickly through short-course RMT therapy combined with orelabrutinib and lenalidomide therapy, thus reducing the physical requirements for patients to tolerate intensive chemotherapy and achieving a certain short-term tumor remission effect and prolongating the survival of patients in this study. Overall, this novel combination regimen has a favorable tumor remission efficacy and survival.

Genetic signature analysis may contribute to individualized targeted therapy (31). As reported, patients with abnormal activation of NF-κB signaling achieved better efficacy (32), which supports our results of the orelabrutinib-containing regimens. Notably, BTK is a crucial regulator of BCR and TLR signaling, especially for PCNSL with NF-κB pathway activation. BTK inhibitors disrupt BCR downstream signaling and induce apoptosis (33, 34). The patients with mutations in all three pathways achieved a CR or PR after treatment, while the patients without mutations achieved a poor response, even PD. In addition, ctDNA monitor is equally important. The blood ctDNA of most patients was negative, and the positive results often indicated the possibility of PD or peripheral invasion. The negative ctDNA after combination therapy indicated that the patient responded to the treatment. Thus, we recommend simultaneous NGS of ctDNA in blood and cerebrospinal fluid. The main reasons are as follows: firstly, the positive rate of ctDNA in CSF is higher; secondly, the gene mutation spectrum detected by next-generation sequencing is broader; thirdly, the recurrence and invasion in and out of CNS can be monitored simultaneously. Moreover, we also found that the gene mutation profiles of CSF were high concordant with that of the tumor sample, which was consistent with the previous study (14).

In the present study, several intriguing cases were observed. Of the 7 RMT prior treated patients continued receiving orelabrutinib and immunochemotherapy therapy, and achieved 4 CR, 2 PR, and 1 PD, suggesting that this combination therapy might induce long-term immune-modulatory effects. For these patients with tumor progression after the RMT regimen, targeted therapy is likely to potentially reduce the burden of tumor and produce the long-term benefit, orelabrutinib has synergistic effects with the immunomodulator lenalidomide. Due to the sample size limitation, findings should be confirmed in future trials with larger data sets.

Regarding the safety analysis, our study demonstrated an acceptable safety profile for the orelabrutinib-containing regimen in patients with r/r PCNSL. Here reported AEs (ie, transaminase increase, fatigue and leukopenia) were mild to moderate and manageable with supportive care. Besides, we found that most AEs were known and also uncommon, and the safety profile are generally similar to lenalidomide/rituximab regimen (35) or HD-MTX/temozolomide/rituximab regimen (36). This implicated that most of these AEs mostly resulted from the rituximab, HD-MTX, or temozolomide alone; and orelabrutinib did not increase the risk of toxicity. In addition, short-course RMT therapy may reduce toxicity in our study. As known, atrial fibrillation, bleeding and infectious complications are common AEs related to BKT inhibitors (37); however, these were not reported in our study, which may be attributed to the high target selectivity and low off-target reactivity of orelabrutinib (18). Besides, no unexpected AEs and grade 4 or more AEs and cardiotoxicity were observed in the present study. Taken together, the orelabrutinib-containing regimen is well-tolerated and manageable in patients with r/r PCNSL.

This preliminary study had several limitations. First, the present results are based on limited sample size and non-randomized retrospective design, which may generate inevitable selection bias. Second, there is a lack of longitudinal CSF sequencing data to elucidate the role of ctDNA of CSF in the treatment monitor and prognosis assessment. Finally, long-term efficacy was not evaluated because of the limited observation period. Thus, strictly designed, large-scale, and high-quality clinical trials should be conducted to validate our findings.

## Conclusions

In conclusion, the high response rate and good tolerance observed in this study suggested that orelabrutinib-containing regimens are a promising therapeutic option for r/r PCNSL. The results also indicated that gene sequencing of tumor specimens can help to screen the patient population responding to BTK inhibitor targeted therapy. The results provide preliminary evidence for the application of orelabrutinib-containing regimens in r/r PCNSL.

## Data Availability Statement

Due to institutional ethics restrictions, the dataset of the patients supporting the current study has not been deposited in a public repository, but is available from the corresponding authors upon request.

## Ethics Statement

This retrospective study was approved by the Medical Ethics Committee of the Beijing Tiantan Hospital (Ethics Approval No. YW2020-038-02) and was conducted in accordance with the principles of the Declaration of Helsinki. All patients provided informed written consent prior to sample collection. The study was exempted informed consent and the protocol was approved by the Ethics Review Committee of Beijing Tiantan Hospital. Written informed consent was obtained from the individual(s) for the publication of any potentially identifiable images or data included in this article.

## Author Contributions

SL and CY conceived conceptualization, performed data analysis, and supervised the project. SL contributed to methodology. YC and XR provided the resources and contributed to project administration. ML, HHJ, KY, SS, MXL, XKZ, XZZ, and QZ performed data curation. CY wrote original draft and involved in writing, reviewing, and editing. All authors contributed to the article and approved the submitted version.

## Conflict of Interest

The authors declare that the research was conducted in the absence of any commercial or financial relationships that could be construed as a potential conflict of interest.

## Publisher’s Note

All claims expressed in this article are solely those of the authors and do not necessarily represent those of their affiliated organizations, or those of the publisher, the editors and the reviewers. Any product that may be evaluated in this article, or claim that may be made by its manufacturer, is not guaranteed or endorsed by the publisher.
