# Supplementary material for: Orelabrutinib Combined With Lenalidomide and Immunochemotherapy for Relapsed/Refractory Primary Central Nervous System Lymphoma: A Retrospective Analysis of Case Series
Source: Front Oncol. 2022 Jun 16;12:901797. doi: 10.3389/fonc.2022.901797 (PMC9243261; doi:10.3389/fonc.2022.901797)
Supplement: Supplementary file 1 [file Table_1.docx]

**Table S1. The treatment regimens and disease progression of each patient.**

| **Id** | **Age** | **KPS** | **Sex** | **Subtype** | **Time to diagnosis** | **Prior line** | **Status** | **Tumor location** | **Prior regimens** | **Treatment regimens** | **Start date of therapy** | **RMT Cycles** | **Time to response** | **Time to best response** | **Best response** | **End date of therapy** | **Follow-up** | **Status (date cutoff)** |
| --- | --- | --- | --- | --- | --- | --- | --- | --- | --- | --- | --- | --- | --- | --- | --- | --- | --- | --- |
| P1 | 56 | 90 | female | non-GCB | 2018/05/28 | 3 | relapsed | Superficial brain | RMT-L | RMT-OL | 2021/02/22 | 1 | 2021/03/30 | 2021/04/29 | CR | 2021/08/10 | 2021/08/10 | PD |
| P2 | 73 | 80 | female | non-GCB | 2020/04/17 | 2 | refractory | Superficial brain | RM | RMT/OL | 2020/10/28 | 3 | 2020/12/21 | 2021/05/31 | CR | ongoing | 2022/03/10 |  |
| P3 | 46 | 60 | female | GCB | 2019/06/20 | 2 | relapsed | Deep brain | RM | RMT/OL | 2021/01/27 | 2 | 2021/02/02 | 2021/06/08 | CR | ongoing | 2022/03/10 |  |
| P4 | 40 | 50 | female | non-GCB | 2019/12/16 | 5 | relapsed | Deep brain | PD-1 | RMT/OL/PD1/Chidamide | 2021/07/29 | 1 | 2021/08/16 | 2021/09/15 | SD | 2021/11/15 | 2021/11/15 | PD |
| P5 | 77 | 50 | male | GCB | 2016/05/04 | 4 | relapsed | Superficial brain | RMT | RMT/OL/PD1 | 2020/11/05 | 1 | 2020/11/11 | 2020/11/11 | PR | 2021/03/16 | 2021/03/16 | PD |
| P6 | 33 | 50 | male | GCB | 2020/01/03 | 2 | relapsed | Deep brain | M | RMT/OL/PD1/etoposide | 2020/12/08 | 5 | 2020/12/22 | 2021/07/20 | CR | 2021/09/28 | 2021/09/28 | PD |
| P7 | 57 | 80 | female | non-GCB | 2021/04/14 | 1 | relapsed | Superficial brain | RMT | RMT/OT | 2021/06/11 | 2 | 2021/07/23 | 2021/11/09 | CR | ongoing | 2022/03/10 |  |
| P8 | 51 | 70 | female | non-GCB | 2021/04/25 | 1 | relapsed | Deep brain | RMT | RMT/OL | 2021/08/03 | 1 | 2021/09/07 | 2021/10/10 | CR | ongoing | 2022/03/10 |  |
| P9 | 63 | 60 | female | non-GCB | 2021/05/20 | 1 | refractory | Superficial brain | RMT | RMT/OT | 2021/07/30 | 2 | 2021/08/03 | 2021/11/30 | PR | 2021/12/14 | 2021/12/14 | PD |
| P10 | 63 | 60 | male | non-GCB | 2021/06/29 | 2 | relapsed | Deep brain | RM | RMT/OL | 2021/12/15 | 2 | 2021/12/21 | 2022/03/10 | CR | ongoing | 2022/03/10 |  |
| P11 | 66 | 80 | male | non-GCB | 2021/10/08 | 1 | refractory | Deep brain | RM | RMT/OL | 2021/12/21 | 1 | 2022/01/21 | 2022/01/21 | CR | ongoing | 2022/03/10 |  |
| P12 | 62 | 40 | female | non-GCB | 2020/12/22 | 1 | relapsed | Deep brain | RMT-L | RMT/OL | 2021/12/22 | 1 | 2022/01/26 | 2022/02/15 | CR | ongoing | 2022/03/10 |  |
| P13 | 78 | 60 | male | GCB | 2021/10/23 | 1 | refractory | Deep brain | RM | RMT/OL | 2021/12/29 | 1 | 2022/01/25 | 2022/02/18 | CR | ongoing | 2022/03/10 |  |
| P14 | 59 | 60 | female | non-GCB | 2021/10/12 | 1 | refractory | Superficial brain | RMT | RMT/OL | 2022/01/07 | 1 | 2022/01/23 | 2022/02/02 | PD | 2022/02/02 | 2022/02/02 | PD |
| P15 | 63 | 90 | female | non-GCB | 2019/12/30 | 2 | relapsed | Deep brain | MT | RMT/OL | 2021/09/08 | 1 | 2021/10/01 | 2021/11/01 | CR | ongoing | 2022/03/10 |  |

GCB, germinal B cell-like; R, rituximab; M, high-dose methotrexate; T, temozolomide; L, lenalidomide; O, orelabrutinib; PD-1, programmed cell death-1.
